# Circadian Rhythms of the Hypothalamus: From Function to Physiology

**DOI:** 10.3390/clockssleep3010012

**Published:** 2021-02-25

**Authors:** Rachel Van Drunen, Kristin Eckel-Mahan

**Affiliations:** 1 MD Anderson UTHealth School Graduate School of Biomedical Sciences, Houston TX 77030, USA; Rachel.VanDrunen@uth.tmc.edu; 2 Brown Foundation Institute of Molecular Medicine University of Texas McGovern Medical School, Houston, TX 77030, USA

**Keywords:** circadian rhythm, clock genes, hypothalamus, extra-SCN hypothalamic nuclei, metabolism, food-entrainable oscillator, obesity

## Abstract

The nearly ubiquitous expression of endogenous 24 h oscillations known as circadian rhythms regulate the timing of physiological functions in the body. These intrinsic rhythms are sensitive to external cues, known as *zeitgebers*, which entrain the internal biological processes to the daily environmental changes in light, temperature, and food availability. Light directly entrains the master clock, the suprachiasmatic nucleus (SCN) which lies in the hypothalamus of the brain and is responsible for synchronizing internal rhythms. However, recent evidence underscores the importance of other hypothalamic nuclei in regulating several essential rhythmic biological functions. These extra-SCN hypothalamic nuclei also express circadian rhythms, suggesting distinct regions that oscillate either semi-autonomously or independent of SCN innervation. Concurrently, the extra-SCN hypothalamic nuclei are also sensitized to fluctuations in nutrient and hormonal signals. Thus, food intake acts as another powerful entrainer for the hypothalamic oscillators’ mediation of energy homeostasis. Ablation studies and genetic mouse models with perturbed extra-SCN hypothalamic nuclei function reveal their critical downstream involvement in an array of functions including metabolism, thermogenesis, food consumption, thirst, mood and sleep. Large epidemiological studies of individuals whose internal circadian cycle is chronically disrupted reveal that disruption of our internal clock is associated with an increased risk of obesity and several neurological diseases and disorders. In this review, we discuss the profound role of the extra-SCN hypothalamic nuclei in rhythmically regulating and coordinating body wide functions.

## 1. Introduction

Most organisms on earth exhibit highly conserved 24 h rhythms in physiology and behavior. Constant 24 h rotations of the earth punctuated by the rising and setting of the sun contribute to an organism’s circadian (i.e., 24 h) biology at the molecular, cellular, and behavioral levels. This internal clock not only sensitizes, but enables an organism to anticipate daily fluctuations in its environment. This time-keeping process operates in almost all cells of an organism and is self-perpetuating, even in the absence of external cues [1]. Although circadian clocks throughout the body are synchronized in large part through the suprachiasmatic nucleus (SCN) of the hypothalamus, rhythmicity in other hypothalamic nuclei has proved to be a critical regulator of physiological rhythms such as the sleep–wake cycle and daily food intake.

**Figure 1:**
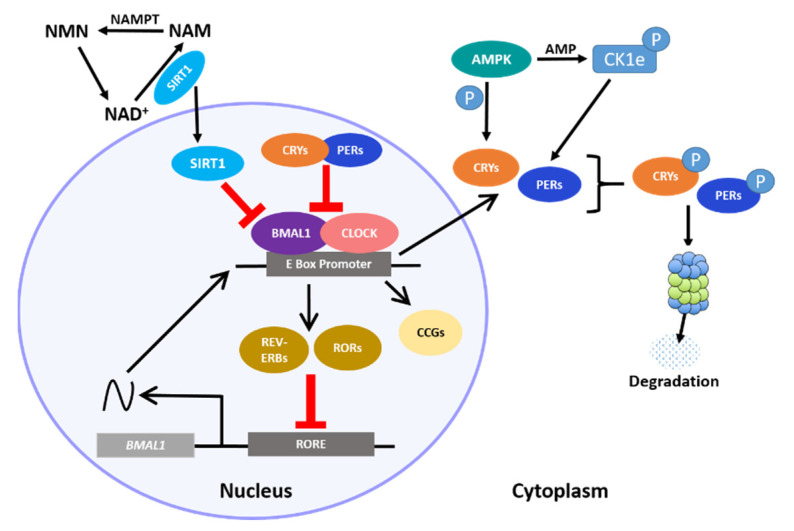
Interactions between the core clock and intracellular metabolism. The heterodimerization of BMAL1 and CLOCK proteins and subsequent binding to E-Box-containing regulatory elements leads to expression of the repressor PER and CRY proteins, the REV-ERBs and RORs, which initiate the auxiliary loop, and the core clock genes (CCGs) that drive numerous other intracellular rhythms. Cytoplasmic PER and CRY proteins are eventually tagged for degradation by AMPK. Rhythmic cellular metabolism, such as rhythmic NAD^+^ abundance, participates in the core clock by direct regulation of clock-associated factors, such as the anti-aging-associated histone deacetylase protein, SIRT1.

The circadian clock in the hypothalamus and elsewhere ultimately depends on 24 h rhythms at the cellular level, where a central transcription–translation feedback loop (TTFL) regulates the expression of key clock transcription factors (TFs). The core loop is a heterodimer consisting of circadian locomotor output cycles kaput (CLOCK) and brain and muscle ARNT-like protein (BMAL1), which promote the rhythmic expression of numerous E-Box-containing output genes, including the Period (Per 1–3) and Cryptochrome (Cry 1–2) genes, which as proteins function as direct repressors of the CLOCK:BMAL1 heterodimer [1,2]. Resumption of CLOCK:BMAL1 activity occurs only when these repressors are degraded by regulators such as the serine/threonine casein kinases (CK1δ and CK1ϵ), which phosphorylate PER, initiating its ubiquitination. CRY turnover is also controlled by phosphorylation; the metabolic sensor 5′ adenosine monophosphate-activated protein kinase (AMPK) tags it for proteasome degradation by direct phosphorylation. Additional loops consisting of the nuclear receptor subfamily 1 D member 1 (NR1D1) also known as REV-ERα/β and the retinoic acid receptor-related orphan receptors (RORs) sustain this core transcriptional loop by transactivating or repressing Bmal1 [3,4,5]. An integral non-circadian loop intertwined with the core clock includes the circadian metabolite nicotinamide adenine dinucleotide (NAD^+^). The NAD^+^-dependent deacetylase sirtuin 1 (SIRT1) directly binds to the CLOCK:BMAL1 heterodimer, and thereby regulates the NAD^+^ salvage pathway transcriptionally [6]. Together, these feedback loops mediate rhythmic expression of hundreds of clock-controlled genes (Figure 1). Importantly, *Bmal1* is the only gene in which single-gene knockout results in full loss of rhythmicity at the cellular and behavioral levels in a normal light–dark cycle [7,8], though double knockouts of *Cry1* and *Cry2* can result in complete arrhythmicity in constant darkness [9,10]. This underscores the robust and resilient, though highly intricate, nature of our internal clocks to maintaining time.

Numerous epidemiological studies have shed light on the importance of rhythmicity on health. Over time, disruption of our 24 h cycle can lead to deleterious physiological outcomes, such as premature aging, and an increased risk for several diseases and disorders including obesity, cardiovascular disease, Alzheimer’s disease and other neurological diseases [11,12,13,14,15,16,17]. Epidemiological studies on night shift workers have revealed a type of desynchrony or “misalignment” of biological processes in individuals under shift work [18]. The circadian mechanisms driving daytime wakefulness find themselves in conflict with the homeostatic drive for sleep that accumulates as daytime progresses, leading to circadian perturbation [19,20,21]. Shift work and jet lag are not the only forms of circadian disruption; nutrient stress (a prominent disruptor of peripheral rhythms [22]), nocturnal light pollution, and mistimed food intake are additional examples of potent *zeitgebers* (or “time-givers”) that alter the internal biological clock and its synchrony across tissues [23,24].

Photic stimulation is the most powerful *zeitgeber* for the brain’s clock. Light is directly received by retinal ganglion cells (ipRGCs), which contain the photopigment melanopsin. The ipRGCs depolarize independently from the rods and cones to relay light information to the SCN, a small region of the anterior hypothalamus with critical synchronizing capabilities. A combination of ablation studies in rats and monkeys, along with clinical psychiatric observations carried out in the mid-1900s first implicated a circadian clock in the hypothalamic region [25,26]. However, it was not until the discovery of the retinohypothalamic tract (RHT) in rats that the SCN was proven to be important for rhythmicity [27]. The SCN was identified in 1972 by two groups, who showed that electrolytic lesion of the SCN in rats resulted in the loss of locomotor and drinking rhythmicity [28,29]. Electrophysiological studies in rats demonstrated that SCN rhythmicity could be maintained for days in vitro following ex-plantation from the surrounding brain tissue [30]. Later studies also revealed that arrhythmicity in hamsters with SCN lesions could be restored when a fetal SCN was grafted onto the lesioned SCN in vivo [31]. Together, these studies underscore the robustness of the SCN and its requirement for circadian rhythms and behavior (reviewed in Herzog et al. 2017 [32]). The SCN coordinates the entire mammalian circadian system, through the complex regulation of electrical and hormonal signals that propagate throughout the brain and the periphery [33]. Though initially thought to be the dominant component of the 24 h biological clock in the mammalian system, genetic editing tools have revealed new roles of the circadian clock throughout various tissues of the body, where it controls processes as disparate as glucose sensitization, fluid balance, immune defense, lipid metabolism, and cell migration, among many others [34,35,36,37,38,39,40,41]. Moreover, when explanted from the body into culture, these tissue clocks maintain rhythms, indicating their own autonomy [42].

## 2. Chronology of Clocks in the Hypothalamic Nuclei

Hypothalamic extra-SCN oscillators are now recognized to play integral roles in essential physiological functions such as eating, sleep–wake cycles, energy metabolism and thermoregulation [43,44,45,46]. One example of diurnal activity considered to be controlled independently of the SCN is food-anticipatory activity (FAA), which can persist in spite of SCN ablation [47]. As the name suggests, FAA involves increased activity in anticipation of an upcoming meal, which is particularly evident when daily feeding is restricted to a temporally restrictive time window. Rats express FAA in constant darkness (or “free-running” conditions) and even in the absence of a functional SCN [47]. These findings point to an elusive food-entrainable circadian oscillator (FEO) which is independent of the SCN [48].

**Figure 2:**
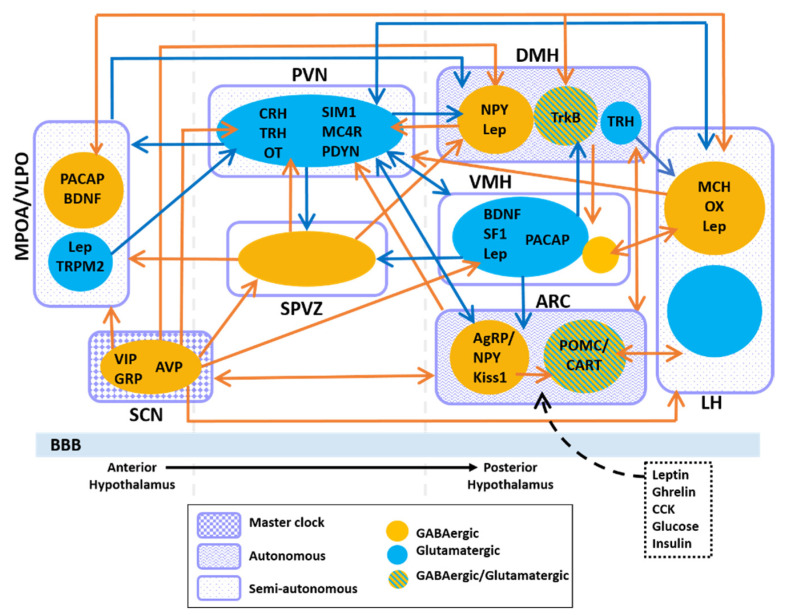
A circuit of hypothalamic oscillators. Numerous afferent and efferent projections characterize the hypothalamic landscape. The “master clock”, or the SCN, projects to the major hypothalamic nuclei while only a few nuclei project back to the SCN. Some regions, such as the DMH and ARC host an autonomous clock while other regions such as the PVN, LH and MPOA/VLPO are more heavily dependent on rhythmic innervations. Clock autonomy for the VMH and SPVZ has not been shown. An array of neural subpopulations in the hypothalamus are sensitive to hormones such as leptin, ghrelin, cholecystokinin (CCK), glucose and insulin, which cross the blood–brain barrier (BBB). These hypothalamic nuclei are often characterized by their inhibitory glutamatergic (blue), or their excitatory GABAergic (orange) projections. The gray dotted vertical lines denote the different regions of the hypothalamus. The general flow of information progresses from anterior to posterior, as indicated by the black arrow. Gastrin-Releasing Peptide; TRH = tyrosine hydroxylase; TRPM2 = transient receptor potential cation channel, subfamily M, member 2; VIP = vasoactive intestinal peptide.

To date, how these extra-SCN oscillators function relative to or in coordination with the SCN is still under investigation. The SCN has direct projections to various hypothalamic regions as well as non-hypothalamic regions such as the periventricular nucleus of the thalamus, the intergeniculate leaflet, the lateral septum and the periaqueductal gray [49]. However, the SCN predominantly innervates hypothalamic nuclei where dense projections to the subparaventricular zone (SPVZ) and the medial preoptic area (MPOA) have been observed [50,51,52]. Neural tracing studies have revealed that the dorsal medial hypothalamus (DMH), arcuate nucleus (ARC), lateral hypothalamus (LH), paraventricular nucleus (PVN), ventral lateral hypothalamus (VMH), and the ventral lateral preoptic area (VLPO) are also targets of SCN projections [50,53] (Figure 2). Collectively, the ARC, VMH, LH and PVN are heavily involved with hunger and satiety as well as metabolic balance [54,55,56,57,58,59,60,61,62,63]. Moreover, neurons in the PVN are also responsible for endocrine regulation through hormone production and release [64,65]. The LH, MPOA, VLPO, DMH and SPVZ regulate sleep/wakefulness, locomotion, and thermoregulation [63,66,67,68,69]. Altogether, these hypothalamic nuclei maintain organism-wide energy balance.

Several years following the discovery of non-SCN-driven circadian rhythms of melatonin release by the retina [70], 27 brain regions were examined for rhythmicity independent of the SCN [71]. Utilizing genetically modified rats harboring a *Period1* promoter-driven luciferase (*Per1-luc*) transgene, real-time bioluminescent recordings of isolated brain tissues cultured in vitro [71] revealed that 14 out of the 27 hypothalamic regions examined were able to maintain autonomous rhythms in *Per1*-*luc* expression [71]. The most robust rhythms were found in the olfactory bulb, the ARC, the pituitary gland and the PVN [71]. Though the SCN is able to maintain rhythmicity over very long periods of time in vitro, non-SCN tissue rhythms dampened much more quickly. This is thought to be due to the loss of synchronization provided by the SCN between individual cells of other hypothalamic nuclei [42]. Nevertheless, these data suggested a role for semi-autonomous extra-SCN oscillators not only in the hypothalamus, but other regions of the central nervous system (CNS) as well.

## 3. A Regulatory Role for Hypothalamic Clocks in Feeding and Food-Anticipatory Activity

A “feeding center” in the mammalian hypothalamus was described almost 70 years ago, based on the variations in food intake upon injury to certain parts of the hypothalamus [72]. Although FAA was described much earlier than this [73], the presence of anticipatory activity leading up to feeding has been reported in a variety of animals including, bees, fish, marsupials, rabbits, weasels and squirrel monkeys [48,74,75]. Meyer-Lohmann conducted a study in 1955 which suggested for the first time that FAA was in fact entrained by a different clock than the one governing locomotor activity. Using mice kept in constant darkness and fed once a day, FAA was maintained at a constant 24 h rate, with free-running activity rhythms shorter than the typical 24 h period [76]. This FAA behavior suggested that two different oscillators were functioning to control different behaviors [76]. Additional evidence provided in 1977 showed that periodic food presentation during the rest phase was actually a stronger entrainer than light in driving rhythmic circadian activity [77]. Collectively, these early findings gave birth to the idea of a “feeding center”, capable of predicting mealtime and highly circadian in nature, through which energy intake could act as a powerful entrainer of the internal biological clock.

The mechanisms and circuits governing feeding are complex, and identifying a precise area for feeding entrainment has been challenging for the field. The activity shift that occurs in response to a narrow time window of food availability usually involves a 2–3 h surge of locomotor activity in advance of food availability, and a phase advance in the release of melatonin and arginine vasopressin (AVP) expression in the SCN [78]. A number of different mouse models have been generated to study the role of the tissue-specific CCGs potentially responsible for FAA. Using the Calcium/Calmodulin-Dependent Protein Kinase II Alpha (*Camk2a*) driver to generate a forebrain deletion of BMAL1 (BKO) in mice resulted in a 90% reduction of *Bmal1* expression in the forebrain region as well as the hypothalamus and the SCN [79]. In this context, weakened rhythms remained in the SCN and the DMH, likely due to remaining rhythmicity in glial cells and unfloxed neurons expressing *Bmal1*. BKO mice showed blunted feeding rhythms and lacked diurnal activity rhythms. Moreover, in constant darkness, the rhythms of the peripheral tissues became desynchronized and dampened. Under restricted feeding (RF), the BKO mice appear to express FAA, while rhythms in the liver and kidney are restored [79]. This BKO model indicates that the FEO can function independent of synchronization in the forebrain. However, using the Nestin promoter (*Nes-Cre*), which is specific to the CNS, Mieda and Sakurai 2011 created *Bmal1^f/fll^;Nes-Cre* (*N-Bmal1^−/−^*) mice which lack BMAL1 throughout the entire nervous system. These *N-Bmal1^−/−^* mice were not behaviorally arrhythmic under constant darkness. However, they were unable to entrain to a RF schedule suggesting that the CNS is still an integral site for the FEO [80]. To specifically examine the role of BMAL1 in extra-SCN hypothalamic regions, the NK2 homeobox 1 driver was used to create *Nkx2.1-Bmal1^−/−^* mice. *Nkx2.1* is a developmental regulatory gene expressed in the posteroventral hypothalamus yet absent in the adjacent domain where the SCN and its neighboring structures develop [81,82]. As a result, the *Nkx2.1*-*Bmal1*^−/−^ mice are devoid of *Bmal1* expression in the preoptic area, the nucleus of the diagonal band, and the majority of the hypothalamus, while *Bmal1* expression is maintained in the SCN [83]. These *Nkx2.1*-*Bmal1^−/−^* mice showed a shifted pattern of night time behavior, with more activity in the second half of the night compared to controls [83]. Though the overall circadian phenotype of these animals was relatively minor, there was a slight increase in rest phase energy intake in *Nkx2.1*-*Bmal1^−/−^* mice compared to littermate controls. Genetically modified mice lacking *Rev-erbα* in *Nestin*-positive cells of the brain showed more remarkable defects in FAA, with a 6 h RF paradigm resulting in almost no FAA in *Rev-erbα-*deficient mice [84]. The findings support the circadian gene *Rev-erbα* as important for neuronal prediction of food availability. Additional studies also highlight *Rev-erbα*’s involvement in various metabolic processes including gluconeogenesis and adipocyte differentiation, noting the gene as a regulator of circadian behavior and metabolism [85]. Moreover, a study by Mang et al. 2016 revealed that *Rev-erbα* knockout mice display an altered sleep homeostasis phenotype, characterized in part by advanced wakefulness relative to the onset of the dark (active) phase, as well as poor adaptability to sleep deprivation [86]. Thus, the altered sleep patterns and inflexibility of the *Rev-erbα* knockout mice may contribute to the notable defects in FAA [86]. Another knockout study targeting the CLOCK paralog, neuronal PAS domain protein 2 (NPAS2), also found that the NPAS2-KO mice displayed altered sleep homeostasis and took substantially longer than their WT littermates to adapt to a RF schedule [87]. While suggestive of circadian genes responsible for FAA, the precise circuits responsible for FAA and the anatomical location of the FEO are still unknown. However, FAA has been shown to induce expression of c-Fos, a marker of neuronal activity, in hypothalamic regions including the ARC, SCN, DMH, PVN, LH and VMH in a manner which follows the degree of caloric restriction. This suggests that one or more of these regions may function together as the FEO and rhythmically modulate feeding [88].

### 3.1. Feeding and the Circadian Clock in Neuronal Subtypes of the ARC

Hypothalamic regulation of feeding begins with the ARC. Positioned lateral to the third ventricle, the ARC forms a complex with the median eminence that is unguarded by the blood–brain barrier (BBB). This unique positioning allows the ARC to sense metabolite, nutrient, lipid and hormone fluctuations in the bloodstream. From this vantage point, the ARC is highly attuned to metabolic and hunger signals to stimulate its first order neuronal subtypes, either the appetite inhibiting pro-opiomelanocortin (POMC)/cocaine amphetamine-regulated transcript (CART) neurons, or the appetite-inducing neuropeptide Y (NPY)/agouti-related protein (AgRP) neurons which function antagonistically to each other. Though the ARC in mice loses the rhythmic expression of clock and GABAergic-related genes when the timing of feeding is restricted to the rest period [89], the ARC still maintains a weak but rhythmic dopamine release when the SCN clock is disrupted [90]. In fact, PER2::LUC recordings reveal endogenous circadian rhythms in an explanted ARC, which can be sustained up to eight days, with higher amplitude peaks in the dorsal ARC versus the lateral ARC [91]. Thus, the ARC appears to have its own autonomous clock. Moreover, it is clear that the intrinsic ARC clock is used to mediate the timing and quantity of food intake. Mice placed on RF display rhythmic changes in AgRP/NPY neuronal activity [92]. When the function of AgRP neurons is ablated in neonatal mice, there is diminished FAA, suggesting that AgRP neurons are involved in FAA [92]. Cre-mediated deletion of *Bmal1* expression in AgRP neurons (ABKO) in adult mice results in increased feeding, daytime hepatic gluconeogenesis, and respiratory exchange ratio in the ABKO mice, although it is unknown whether these mice express FAA [93]. Lesioning of NPY-ARC neurons causes the uncoupling of feeding from sleep–wake cycles in rats; however, it does not result in loss of food predictability under RF [94]. Similarly, using saportoxin conjugated to leptin to eliminate ARC leptin neurons disrupts the rhythmic integration of activity and body temperature while leaving FAA unaltered in rats [95]. These studies indicate that the FEO is not solely dependent on the ARC.

Using transcriptomics to analyze appetite-regulating pathways in AgRP and POMC neurons of the hypothalamus has revealed an enrichment of circadian signaling factors during food deprivation [96]. Other studies have also revealed changes in the oscillation patterns of clock genes in ARC neurons in response to meal timing [97]. In addition, the neurons of the melanocortin system, predominately localized to the ARC, are well-established contributors to the hypothalamic regulation of metabolism and potential regulators of glucose homeostasis [98].

There are a few crucial molecular signals and receptors in the ARC that are essential to mediating feeding and appear to be involved in FAA. A recent study identified a circadian role for the neurotrophin receptor p75NTR activity in AgRP neurons in driving FAA [99]. p75NTR is a brain-derived neurotrophic-factor (BDNF) receptor that rhythmically mediates oscillation of certain metabolic liver genes [100]. Mice lacking p75NTR in either the AgRP neurons or the entire brain express FAA only during the active phase, but not the rest phase [99]. Additionally, the mammalian target of rapamycin (mTOR) protein, highly expressed in the ARC and PVN, is implicated in rhythmic feeding [101,102]. mTOR is a nutrient-activated serine-theronine kinase which regulates cell growth and metabolism. Activation of hypothalamic mTOR signaling in mice results in anorexia and substantial weight loss [102]. Interestingly, the knockout of mTOR complex 1 (mTORC1) dampens the rhythmic expression of AgRP and NPY, but is negligible for the regulation of feeding and energy homeostasis [103]. However, in POMC neurons, the mTORC1 signaling pathway is necessary to carry out leptin-mediated suppression of food intake [104]. Together, these findings suggest that molecular signals within the ARC mediate the timing of food consumption, but may also potentially function as part of the FEO.

### 3.2. Feeding and the Circadian Clock in Neuronal Subtypes of the LH, PVN, DMH and VMH

The function of the LH is best summed by the question “*to rest or ingest?*” The LH tightly controls energy expenditure and food intake to maintain energy homeostasis [105]. In particular, the abundant orexin (OX) neurons of the LH are crucial for feeding and wakefulness and are sensitized to promote arousal, feeding, locomotion and drinking in the face of energy deficits [106,107,108]. Widespread projections to the autonomic nervous system (ANS) and the SCN as well as interactions with the neuroendocrine system makes the LH an ideal candidate for involvement in FAA and the FEO. In a RF experiment with a two-hour feeding window, lesions of the LH attenuated FAA behavior as measured by locomotion; however, LH-lesioned rats still show some FAA [109]. However, mice under RF show increased activity of OX-LH neurons in anticipation of feeding [110], suggesting some contribution of the LH in FAA.

Relatively recent research has begun to more thoroughly examine the PVN from a circadian perspective. Inhibitory GABAergic projections from the ARC innervate the PVN, a hypothalamic nuclei that functions as an integrator of metabolic, neuroendocrine, and satiety signals with direct inputs to the ANS. Owing to its diverse functions, the PVN contains a variety of neural subtypes which both receive and send out projections from neighboring hypothalamic nuclei including the DMH, VMH, LH, SCN, MOPA, and SPVZ [111,112]. Ablation of single-minded homolog 1 (SIM1)-positive neurons, a marker of PVN neurons, leads to hyperphagia (over-eating) and obesity [113]. Abe et al. 2002 demonstrated that the PVN maintains 92% rhythmicity following excision from brain tissue suggesting that a semi-autonomous clock is present [71]. However, other studies centered on the examination of SCN grafts reveal that the PVN is still heavily dependent on the SCN for rhythmic activity [114]. A prime example of this includes a recent optogenetic study, which identifies a SCN–PVN–LH neurocircuit involved in the regulation of wakefulness [115]. These data suggest that the cortisol-releasing hormone (CRH) neurons in the PVN may act as a “pulse generator”, whose excitability is regulated by SCN innervations [115].

To determine a potential role for the PVN in FAA, mice with PVN lesions were tested for FAA activity [109,116,117]. In spite of its known role in energy intake, lesion of the PVN did not disrupt FAA [118]. In spite of these findings the PVN still presents an attractive possibility for being part of the FEO. For example, oxytocin (OT) has recently been postulated as the main hormonal cue for FAA due to findings linking food entrainment in rabbits to OT-PVN neurons [119]. Although the SCN has direct projections to the PVN, the PVN neuroendocrine neurons are also indirectly modulated by the SCN via its innervation of the DMH, ARC and MPOA [120,121].

SIM1-PVN neurons can be divided into two satiety-sensitive subtypes, melanocortin receptor 4 (MC4R) and prodynorphin (PDYN) neurons [122]. Ablation of these neuronal subtypes leads to hyperphagia, obesity, and reduced energy expenditure [123,124]. PVN neurons act as secondary order neurons receiving direct projections from the primary order neurons of the ARC. Therefore, ARC-PVN projections are vital to regulating food intake. Rats under a narrow four-hour daily RF window showed elevated NPY levels in the PVN prior to feeding time [125]. ARC-mediated NPY release in the PVN is therefore likely an important orexigenic signal for FAA. From another perspective, the SCN’s projections to the PVN are also important to feeding through a light-coupled pathway. During the daytime, light exposure suppresses food intake and increases cFos expression in AVP-SCN neurons and OT-PVN neurons in mice [126]. Retrograde tracing has revealed that AVP-SCN neurons terminate on OT-PVN neurons, while blocking OT neurons eliminates the light induced suppression of food intake [126]. These findings highlight a potential neurocircuit explaining light induced suppression of food intake.

One of the main initial contenders for the FEO was the DMH [127]. Gooley et al. 2006 reported that DMH lesions in rats resulted in loss of FAA as well as disrupted body temperature and wakefulness, and that the degree of food entrainment corresponded to the number of DMH cells remaining post-lesion [128]. Subsequent studies showed that expressing *Bmal1* in the SCN of *Bmal1^−/−^* mice resulted in a restoration of light-entrainable but not food-entrainable circadian rhythms [129]. Injection of the *Bmal1* vector to the DMH restored food-entrainable but not light-entrainable circadian rhythms, suggestive of a possible role of the DMH as the site of the FEO [129]. However, follow-up studies contested these findings, rendering the DMH’s contribution to FAA inconclusive at best [130,131]. Specifically, robust FAA has been found in rats with lesioned DMH [132], though original results obtained by Gooley et al. 2006 differed in several key areas: food type, lesion method, food locations, and cage configuration, among others. Cage configuration has been ruled out as a major factor [130].

Interestingly, the DMH has reciprocal feedback to the SCN whereby it can suppress SCN activity, leading to increased locomotor activity during the rest phase in mice [133]. One potential mechanism for DMH suppression of homeostatic feeding is through BDNF tropomyosin receptor kinase B (TrkB) receptor-expressing DMH neurons [134]. Mutation of the *Bdnf* or *TrkB* receptor genes leads to obesity both in humans and mice [134]. TrkB-expressing DMH (DMH^TrkB^) neurons are also activated post-refeeding or during overnight fasting [134]. Chemogenetic activation of DMH^TrkB^ neurons elicits the suppression of food intake during the active phase, whereas chemogenetic inhibition of the same neurons leads to food intake only during the rest phase [134]. However, one study revealed that optogenetic stimulation of inhibitory GABAergic DMH neurons projecting to PVN in particular actually promote food intake [135]. Collectively, these data suggest that the DMH is still an integral player in the circadian regulation of energy intake, though its contribution to FAA is less clear.

Finally, the VMH has also been postulated to contribute to FAA. The VMH is innervated by multiple hypothalamic regions including the ARC, DMH and LH [136,137], and is characterized by steroidogenic factor 1 (SF1) neurons that are highly sensitive to glucose and leptin [138,139,140]. Lesions of the VMH do not completely inhibit FAA; although under TRF, mice with VMH lesions display increased arousal-associated with FAA [141]. Five to nine weeks following the VMH lesion surgery, Mistlberger et al. 1984 reported FAA was dampened or absent in VMH lesions rats; however, recovery of FAA was seen during a second phase of RF 14–21 weeks after the surgery [142]. These findings indicated the VMH is not ultimately essential for FAA.

**Figure 3:**
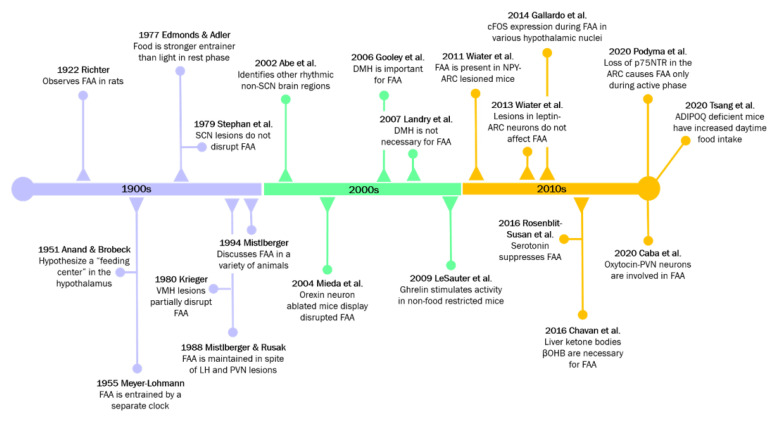
Tracking the “food-entrainable oscillator” through time.

Collectively, studies involving numerous nuclei of the hypothalamus suggest that the FEO may not be localized to one region, but rather dispersed among CNS and peripheral components, with one or more components residing in the hypothalamus to coordinate or respond to peripheral energy status [143,144,145]. One notable study found that in mice with liver-specific deletion of *Per2*, FAA was almost completely abolished [146]. Interestingly, using RNA sequencing, researchers identified the liver-derived ketone body, β-hydroxybutyrate (βOHB) as essential for driving FAA in mice [146]. The restoration of FAA from these liver-specific mutant mice by the administration of exogenous βOHB suggests that the liver may also be a fundamental part of the FEO. The influence of peripheral energy states on hypothalamic regulation of rhythmic feeding has also proved to be important reciprocal information. For example, the adipokine adiponectin (ADIPOQ) is released from adipose tissue, and regulates diurnal feeding rhythms through hypothalamic clocks [147]. *Adipoq*-deficient mice display augmented daytime food intake which is further reinforced by the disrupted expression of core clock gene and appetite-associated hypothalamic genes in the ARC and DMH [147]. For a rough timeline of mechanistic insights, see Figure 3.

## 4. Involvement of the Intrinsic Hypothalamic Clocks in Metabolic Regulation

Metabolism is inextricably linked to circadian rhythms. High-throughput gene expression analysis has revealed that approximately 5–15% of the transcriptome shows circadian rhythmicity across a variety of peripheral tissues [148]. Many of these rhythmic genes are involved in important metabolic pathways, such as glucose and lipid metabolism, and oxidative phosphorylation. Genetic animal models of circadian disruption support a direct role of the circadian clock genes in metabolic regulation. For example, mutation of the circadian genes *Ck1* in Syrian hamsters and the Fbx13/21 gene ortholog in mice both result in shortened period lengths and increased energy expenditure in mice [149]. In peripheral tissues, liver-specific deletion of *Bmal1* leads to fasting-induced hypoglycemia, hypoketosis and impaired mitochondrial fatty acid oxidation [150]. In contrast to the changes seen in mice with *Bmal1* deletion in the liver, Pdx1-Cre mice crossed with *Bmal1^fl/fl^* mice (to produce mice lacking *Bmal1* in pancreatic tissue) show hypoinsulinemia and hyperglycemia following food intake [151]. Thus, the contrasting effects of clock controlled tissue-specific functions on metabolism underscores the necessity for a higher order control system to integrate and balance these contradicting actions of the circadian clock.

Changes in metabolism are frequently observed by specific alterations in adipose tissue. Two main types of adipose tissue exist in mammals: white adipose tissue (WAT) and brown adipose tissue (BAT). WAT is essential for energy storage while BAT is important for energy burning, otherwise known as thermogenesis [152,153]. Stimulated by cold conditions, BAT is capable of converting the stored triglycerides from WAT into heat to maintain body temperature in an organism [154]. Both adipose types are heavily innervated by the SNS whose projections stem from hypothalamic regions including the ARC, MPOA and the PVN [155,156]. Thus, the hypothalamic nuclei utilize their WAT and BAT downstream targets to help maintain energy balance.

### 4.1. Circadian Regulation of Metabolism by the ARC

The ARC plays a crucial role in hypothalamic metabolic regulation. A majority of the AgRP neuron population reside in the ARC, which communicates directly and indirectly with the SCN [112,157]. SCN projections to the ARC contribute to the ARC’s sensitivity to glycemia [158]. Lesion or electrolytic ablation of the SCN alters glucose metabolism and impairs the robust circadian firing of POMC α-melanocyte-stimulating hormone (α-MSH) ARC neurons, suggesting that some of ARC rhythmicity is derived in part from SCN communication [159,160]. The reciprocal projections are also important; when the ARC to SCN projections are severed in rats, rhythmicity in locomotion, corticosterone (CORT) and body temperature is lost under constant darkness [161]. This indicates that ARC rhythmicity is important in synchronizing body wide functions. Multiple studies using either brain slice cultures from transgenic mice or *Per1/Per2* in situ hybridization have shown the ARC has its own diurnal rhythmicity expressed by clock genes [71,162]. A recent study using the forebrain-specific BMAL1 KO mice revealed a cell autonomous clock in hypothalamic AgRP neurons, which specifically controls glucose production during fasting and energy intake during the active phase [163]. ARC regulation of energy expenditure occurs via the ANS which indirectly projects through the PVN, VMH and LH [157,164]. Leptin fluctuations stimulate AgRP and POMC ARC neurons whose firing through hypothalamic intermediaries stimulate changes in heart rate, blood pressure and hepatic insulin sensitivity [165,166]. A crucial ARC neural subtype is the Kisspeptin-expressing neurons (Kiss1) which upon silencing leads to bodyweight gain in mice with dysregulated rhythms in feeding, sleep and body temperature without affecting the circadian expression of the SCN [57]. Dense projections of the Kiss1-ARC neurons to the SPVZ and DMH regions along with wiring to AgRP and POMC neurons in the ARC suggest that the Kiss1-ARC neurons can also converge on these SCN targets to rhythmically regulate their output [57]. An intriguing ARC-neural subtype from a circadian standpoint are pituitary adenylate cyclase-activating polypeptide (PACAP) neurons, PACAP is well known to modulate light-induced phase resetting in the SCN, and PACAP expression neurons of the ARC can inhibit food intake [167] through a POMC-dependent mechanism [168].

**Figure 4:**
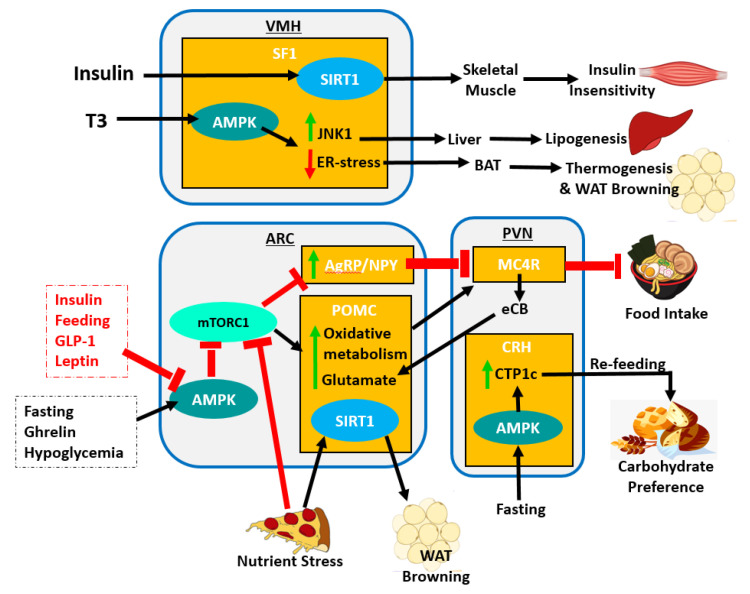
Organism-wide interactions with hypothalamic nuclei alter metabolism and energy homeostasis. AMPK activity is inversely affected by fasting, ghrelin and hypoglycemia compared to insulin, feeding, GLP-1 and leptin. Inhibition of mTORC1 by AMPK increases *Agrp*, in turn inhibiting MC4R neurons, and promoting energy intake. Alternately, inhibition of AMPK allows mTORC1 activity, which promotes *pomc* expression, oxidative metabolism, and an increase in activation of the MC4R-PVN neurons that signal satiety. In addition, the activation of MC4R neurons stimulates production of eCB, which feedback to POMC neurons to increase glutamate production, thereby increasing glutamatergic POMC neuronal input to the PVN. Nutrient stress is also a strong inhibitor of mTORC1 activity and a stimulator of SIRT1 expression in POMC-ARC neurons, whose downstream projections initiate WAT browning. Fasting results in AMPK activity in CRH-PVN neurons, resulting in a carbohydrate preference during re-feeding. Other nutrient signals such as insulin also stimulate the SF1-VMH neurons to express SIRT1 that increases insulin insensitivity in skeletal muscle. The thyroid hormone, T3 elicits AMPK activity in the SF1-VMH neurons and through a dual pathway that increases JNK1 and decreases ER stress to produce hepatic lipogenesis and BAT thermogenesis/WAT browning, respectively.

At the molecular level, AMPK activity results in the activation of *Agrp* expression and the inhibition of *Pomc* expression through regulation of mTORC1 pathway. AMPK inhibits mTOR signaling preventing mTORC1-mediated oxidative metabolism in POMC neurons which inhibit food intake [104] while also promoting *Agrp* expression which is otherwise inhibited by the mTOR pathway [169]. Various factors such as insulin, feeding, glucagon-like peptide-1 (GLP-1), and leptin inhibit AMPK activity in the ARC. However, other signals such as ghrelin, hypoglycemia and fasting trigger AMPK-ARC activity (Figure 4). The deletion of SIRT1 from POMC neurons results in increased propensity to develop obesity, specifically in female mice under nutrient stress [170]. Researchers further show decreased energy expenditure due to impaired sympathetic nerve activity in WAT [170]. SIRT1 overexpression in POMC neurons, protects against age-associated weight gain, adipose expansion, and reduced energy expenditure [170]. It is clear from these studies that the ARC has an essential role in rhythmic regulation of body wide metabolism.

### 4.2. Circadian Regulation of Metabolism by the PVN

The PVN lies downstream of the ARC as an integrator of metabolic, neuroendocrine, and satiety signals. Innervated by inhibitory GABAergic projections from the ARC, the excitatory glutamatergic neurons of the PVN terminate on several regions, including the nucleus tractus solarius (NTS) and the parabrachial nucleus (PB) [171,172,173]. Though its role in energy intake is well accepted, only recently has circadian rhythmicity in the PVN been shown to be a critical regulator of rhythmic energy intake and metabolism. Recently, diurnal variations in the firing of PVN neurons has been observed. Deletion of *Bmal1* expression from the PVN of mice inhibits this diurnal firing, leading to reduced diurnal rhythmicity in metabolism [174]. Interestingly, BMAL1 controls the rhythmic expression of GABA-A receptor γ2 subunit, and the absence of diurnal γ2 rhythms results in loss of diurnal metabolism. Thus, the dynamic responsiveness of BMAL1-driven PVN neurons to GABAergic neurotransmission is essential for mediating diurnal metabolism [174].

Upon activation, the MC4R-PVN neurons signal satiety to decrease food intake. The recent study by Mazier et al. 2019 reports a potential molecular pathway for PVN-mediated satiety [175]. mTORC1 blocks the synthesis of endocannabinoids (eCB) by the PVN neurons; however, AMPK-mediated inhibition of mTORC1 enables eCB production to resume in the PVN. Binding of eCB to the cannabinoid receptor 1 (CB1R) on target POMC-ARC neurons causes increased glutamate release and thus increased glutamatergic input to the PVN eliciting reduced food intake [175]. Identifying involvement of mTORC1, which is closely associated with TTFLs in the core clock mechanisms suggests a molecular basis for rhythmic activity of PVN neurons and, therefore, PVN-mediated circadian regulation of metabolism.

### 4.3. Circadian Regulation of Metabolism by the VMH, DMH and LH

The VMH has been well known for nearly a century for its essential regulation of metabolism [176]. In 1980, Ishikawa and Shimazu found that bilateral electrolytic lesions of the VMH, but not the SCN, substantially reduced the hepatic rhythms of glycogen metabolism in rats fed during the active phase [177]. This implicated a greater role of VMH in rhythmic metabolic homeostasis. Although no overt PER2::LUC rhythms have been observed in explanted VMH organotypic slices [91], hypothalamic lesions of the VMH are known to dramatically increase food consumption, reduce energy expenditure, and induce hyperglycemia and obesity [178]. Knockout of *Vglut2* in SF1-VMH neurons results in hypoglycemia in mice [179]. Using mice with *Bmal1* ablation in the SF1-VMH neurons, Orozco-Solis et al. 2016 found that these mice displayed augmented BAT activity during their active phase, leading to increased body temperature, increased energy expenditure, and reduced body weight [180]. Like the ARC, the VMH contains PACAP-expressing neurons, and PACAP-VMH neurons are sensitive to energy status and involved in glucose homeostasis [181]. Blocking PACAP signaling leads to attenuated leptin-induced hypophagia and hyperthermia [182] indicating that these PACAP-VMH neurons are key mediators of leptin-regulated energy homeostasis.

Interestingly, the liver-derived endocrine hormone fibroblast growth factor 21 (FGF21), which signals starvation to modulate fuel partitioning and metabolism, has substantial effects on the peripheral tissues as well as the CNS to regulate circadian behavior and metabolism [183]. FGF21 can transverse the BBB to directly activate excitatory glutamaterigic VMH neurons eliciting suppressed sugar and carbohydrate intake and increased energy expenditure [184]. FGF21 also affects calcium signaling in glucose-sensitive VMH neurons, resulting in increased glucose sensitivity [184]. Thus, the diurnal expression of this hepatic-derived hormone regulated by lipid metabolism has the ability to diurnally affect specific VMH neurons. Another circadian pathway linked to BAT thermogenesis and energy expenditure begins with the rhythmically oscillating thyroid hormone triiodothyronine (T3) which was identified as an activator of the AMPK pathway in SF1-VMH neurons [185]. AMPK activity in SF1-VMH neurons simultaneously decreases ceramide-induced endoplasmic reticulum stress to promote BAT thermogenesis, while also increasing c-Jun N-terminal kinase (JNK) pathway activation to promote hepatic lipid metabolism through PNS and SNS innervations from the VMH [185]. While AMPK evidently plays a central role in VMH function in the periphery, the presence of the deacetylase SIRT1, stimulated by insulin, is also crucial for normal VMH function. For example, mice lacking SIRT1 are hypersensitive to dietary obesity [186]. Contrastingly, overexpression of SIRT1 in the SF1-VHM neurons render mice more resistant to diet induced obesity due to increased energy expenditure and improved insulin sensitivity in skeletal muscle [186]. These intriguing findings suggest that the non-autonomous clock of the VMH has an important role in rhythmic energy expenditure through regulation of BAT activity.

Sitting dorsal to the VMH and populated by NPY and α-MSH terminals, the DMH is another primary target of ARC, SCN and SPVZ innervation. Dense DMH projections to the VLPO, the locus coeruleus, and the OX-LH neurons are thought to mediate sleep–wake cycles [91,187,188]. Furthermore, a plethora of afferent and efferent connections between hypothalamic sites involved in blood pressure, feeding, metabolism, and thermoregulation, renders the DMH essential to the integration of hypothalamic circadian rhythms [66,91,187]. Though Per2::LUC rhythms support the presence of a semi-autonomous oscillator in the DMH, the oscillator dampens more rapidly than that of the ARC [91]. Excitotoxic lesions of the DMH have been shown to attenuate locomotion, feeding, wakefulness and most substantially serum CORT levels [66]. It is clear that energy status strongly controls DMH activity, as it receives visceral afferent signals from the PB and responds to feeding-related peripheral hormones such as leptin, ghrelin and CCK [98]. Selective activation of the DMH leptin-sensitive (DMH^LepRb^) neurons by pharmacosynthethic receptors increases BAT thermogenesis and locomotion, resulting in weight reduction without affecting food intake [189]. Moreover, CRE-mediated deletion of DMH^LepRb^ neurons produces an opposite effect, highlighting a role for the DMH^LepRb^ neurons in controlling energy expenditure [189]. A similarly powerful diurnal hormone is GLP-1 produced both by the intestinal cells and the NTS neurons, of which the GLP-1-NTS neurons synapse onto the glutamatergic DMH neurons [190]. Injections of GLP-1 into the DMH increases BAT thermogenesis, while knockdown of the GLP-1 receptor in the DMH increases adiposity, attenuates energy expenditure and BAT thermogenesis, and increases insulin resistance [190]. Both leptin and GLP-1 are secreted in response to nutrient intake, thus these hormones connect the *zeitgeber* of food intake to the metabolic clock in part through their interactions with the DMH.

The LH is an extensively interconnected region which consists of numerous distinct neural subtypes such as glucose-sensitive OX neurons which are activated in response to insulin-induced hypoglycemia [191]. Though known for its role in diurnal feeding anticipation and wakefulness, the LH’s contribution to metabolic regulation from a circadian standpoint has not been fully elucidated. Notably, unlike the PVN whose destruction produces obesity, the destruction of the LH instead results in anorexia [72,192]. Viral tracing experiments reveal bidirectional projections between the ARC, VMH and PVN, indicating interconnectedness in crucial metabolic regions [193,194]. Moreover, the OX-LH neurons can regulate BAT thermogenesis to effect overall energy expenditure [195,196]. GLP-1 receptors are also present in the LH and are critical for controlling food intake and bodyweight [197]. In all, the LH’s diverse inputs and neural subtypes suggests further roles in rhythmic metabolic homeostasis that are yet to be discovered.

## 5. The Emerging Regulatory Role of Hypothalamic Clocks in Thirst Anticipation and Fluid Balance

Since organisms have evolved strong systems to balance metabolism and anticipate feeding, it is reasonable to expect that organisms have also evolved robust methods to anticipate thirst. Mammals in particular experience continual water loss through evaporation of hypotonic fluid during breathing or sweating and through excretion of urine. Thus, maintaining a body fluid balance is reliant on instinctive processes to ensure fluid intake is carried out. In the 1950s, a series of studies were conducted wherein salt was infused into the brains of goats with the hopes of uncovering an osmosensor in the brain responsible for thirst [198,199]. These experiments were successful in revealing a small region known as the subfornical organ (SFO), which lies just outside of the BBB and is capable detecting blood osmolarity. Similarly, the hypothalamic region of the organum vasculosum lamina terminalis (OVLT) also lies outside the BBB to sense blood osmolarity. The SFO and OVLT integrate neural signals and share bidirectional projections with the MPOA to maintain fluid balance; together these nuclei make up the lamina terminalis (LT). The SFO sends glutamatergic projections to the PVN to mediate secretion of AVP [200], and possibly to modulate blood pressure and heart rate via the PVN-SNS [201]. Interestingly, AVP-secreting SCN neurons project to the OVLT to mediate the anticipatory thirst prior to sleep in mice [202]. Later during sleep, AVP-SCN neurons appear to stimulate AVP release by the OVLT, which would trigger renal water retention [203]. This brief surge in drinking prior to sleep in mice results in small 1–2% fluctuations in plasma osmolarity that would otherwise occur during sleep [202]. Thus, the SCN may help prevent dehydration during sleep and later during sleep promote water retention [204]. The presence of an autonomous circadian clock has been reported in the SFO. Studies utilizing the PER2::LUC mice have revealed that the SFO and the OVLT are strongly rhythmic, and can maintain rhythms for up to 21 days following explanation and forskolin treatment, and possess intrinsic circadian timekeeping properties [205]. These findings suggest that the SFO and OVLT may have greater circadian contribution than previously expected to the daily regulation of thirst and fluid balance.

## 6. The Circadian Link between Metabolism, Obesity and Sleep

As important the role of the hypothalamus is in energy intake, thirst anticipation and metabolic balance, the hypothalamus also plays an essential role in sleep onset and maintenance. Severe ramifications can result from sleep disruption as exemplified by the deleterious effects of chronic jet lag or shift work in humans. Severe sleep disruption in rodent models can even result in death [206]. One study on sleep deprivation found that in normal healthy adults, sleep deprivation led to decreased leptin levels and increased ghrelin and hunger [207,208]. Interestingly, clinical studies suggest a correlation between sleep time and body mass index [209]. Furthermore, clinical trials implementing shortened sleep durations in subjects have been connected to reduced energy expenditure, which affects thermogenesis and can attenuate rhythmicity in core body temperature [210,211]. This is likely due in part to changes in galanin- and GABA-producing neurons of the preoptic area, the activation of which in mice increases sleep, but results in a drop in core body temperature [212]. The disruption of sleep during certain phases of the sleep cycle is also postulated to be connected to metabolic function. When subtle tones are used to disrupt slow wave sleep without waking the subject or shortening sleep time, researchers find a corresponding decrease in the subject’s glucose tolerance [213]. Moreover, the hypothalamus lies as the central coordinator of sleep patterns, via the SCN projections to the PVN which controls the production and secretion of the rhythmically oscillating sleep hormone melatonin in the pineal gland [214].

Multiple studies have begun to dissect the interconnectedness between hypothalamic control of circadian signaling, metabolic energetics, and sleep–wake cycles [215,216]. Two neuron types predominately regulate wakefulness in the hypothalamus: the monoaminergic neurons of the posterior hypothalamus and the OX neurons in the LH. Knockout of Orexin receptor 2 in mice elicits increased susceptibility to obesity [217]. More intriguingly, OX-LH neuronal activity is modulated by glucose [106,218]. Findings from Borniger et al. 2018 further support a linkage between glucose homeostasis and sleep regulation by OX neurons. In a mouse model of breast cancer, tumors were found to deregulate satiety hormones, altering OX neuronal activity, and leading to impaired sleep and glucose metabolism [219]. The VLPO region appears to be involved in sleep induction and maintenance [220]. During wakefulness, these neurons are inhibited by norepinephrine from the locus coeruleus, but non-rapid eye movement sleep (NREM) involves a decline in norepinephrine signaling, and an induction of GABA release from VLPO nucleus neurons. Glucose contributes to sleep onset by exciting sleep-promoting neurons in the VLPO [221]. This potentially corresponds to the sleepiness that creeps up following a meal. Interestingly, though the SCN is always active during the light cycle in diurnal and nocturnal species, the VLPO is always active during the sleep cycle in diurnal and nocturnal species. Though the SCN does have some projections to the VLPO and orexin neurons of the hypothalamus [66], these are not sufficient to maintain rhythmicity in the sleep–wake cycle, and rather the projections to the SPVZ neurons are required [222]. The DMH, which receives heavy innervation from the SPVZ, is thought to integrate circadian signals to regulate sleep and wakefulness [66,223]. Recently, SCN projection to CRH-PVN neurons have also been shown to contribute to wakefulness [115], highlighting how much still remains to be uncovered in the hypothalamic role of sleep regulation, circadian rhythms and metabolism.

## 7. Circadian Regulation of Metabolism by Neuroendocrine Hormones

**Figure 5:**
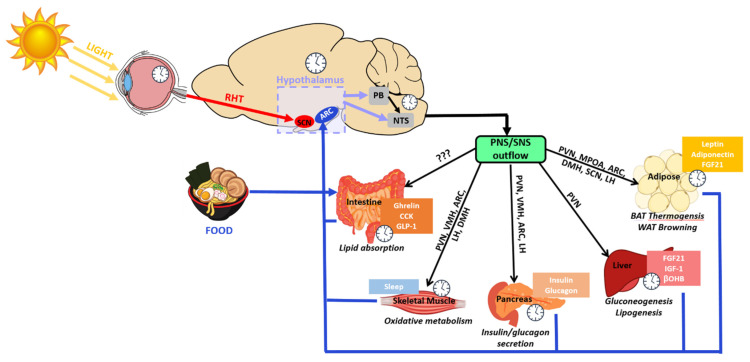
Hypothalamic-mediated circadian regulation of physiological functions. Powerful *zeitgebers* such as light and food have extensive effects on the circadian activity of cellular and tissue functions. The light-sensitive ipRGCs in the retina send signals to the master clock, the SCN, via the RHT. Food consumption stimulates various metabolic and digestive mechanisms which produce a variety of molecular signals and hormones. Nutrient-sensitive neurons in the circadian-regulated hypothalamus pick up the fluctuations in nutrients and hormones and relay the information downstream to the PB and the NTS which regulate the function of tissue processes through the parasympathetic and the sympathetic nervous system (PNS/SNS). With the exception of the intestines, which currently have not been found to have direct innervation from any hypothalamic nuclei, many metabolic-regulating tissues have direct or indirect innervations from various hypothalamic nuclei.

Metabolic homeostasis is predominantly synchronized by the hypothalamic clocks. However, reciprocal relationships between the hypothalamus and peripheral endocrine organs are also required (Figure 5). For example, physiological fluctuations triggered by external stressors, such as changes in blood pressure or glucose levels, can influence SCN neuronal behavior [224]. The sympathetic and parasympathetic branches of the ANS feedback to hypothalamic areas, namely the PVN, which then may indirectly relay the information to the SCN. Furthermore, nutrients and hormones that cross the BBB will be sensed by the ARC-ME complex, and relayed to upstream hypothalamic areas such as the PVN. This is particularly important for the rhythmically fluctuating hunger and satiety hormones, such as leptin and ghrelin. Adipose-derived leptin activates NPY/AgRP and POMC neurons to suppress food intake and stimulate energy expenditure [225]. Leptin also stimulates fatty acid oxidation in skeletal muscle, promotes the uptake of glucose, and improves insulin sensitivity via central and peripheral mechanisms [226,227]. Expression of leptin receptors in POMC neurons of morbidly obese, diabetic and leptin receptor-deficient mice (*Lepr^db/db^*) results in a reduction in energy intake accompanied by normalized glucose levels and attenuated body weight gain [228]. Interestingly, leptin production in WAT is sufficient to drive diurnal oscillations in circulating leptin, independent of feeding. Adipose-specific ablation of *Bmal1* does alter the energy regulation of mice as reflected by reduced levels of triglyceride and polyunsaturated fatty acids circulating through the hypothalamus [190]. In addition, the SCN potentiates the response of ARC neurons to circulating leptin to maintain long-term homeostasis in energy balance [229].

### 7.1. Cortisol-Releasing Hormone (CRH)

A range of neuroendocrine hormones are produced by several hypothalamic nuclei and circulated by secretion into the third ventricle. This includes the well-known oscillatory glucocorticoid hormone, CORT, which is produced by PVN corticotrophin-releasing hormone (CRH) neurons and is critical in the hypothalamic–pituitary–adrenal (HPA) stress axis. Although typically associated with stress, CORT, has been shown to reflect variations in metabolic rate independent of psychological stress [230]. CORT circulation is highly rhythmic, returning to the brain through the ARC. ARC detection of CORT also follows a circadian pattern, dependent on target glucocorticoid and mineralocorticoid receptors in the ARC [231]. Prolonged elevation of glucocorticoid levels in mice results in overconsumption of food via inhibition of CRH-expressing neurons, lowering CORT levels and stimulating NPY expression [232,233]. Additional studies have revealed that both NPY and AgRP expression are differentially responsive to stress via direct innervation from PVN-CRH neurons to the ARC [234,235,236]. NPY neurons of the ARC innervate the PVN, resulting in NPY-mediated CORT production, food intake, and increased PVN activity [237,238]. NPY injection in the PVN of mice results in BAT thermogenesis and increased WAT lipoprotein lipase enzymatic activity [55]. Microarray analyses reveal that rhythmic NPY neurons in part control circadian transcriptional activity in the mouse liver [239]. These findings underscore a bidirectional relationship between the HPA axis and rhythmic ARC and PVN neurons, suggesting an important feedback loop whereby stress-induced chronodisruption influences metabolism and food intake. The recent identification of a neurocircuit whereby GABAergic SCN neurons project to CRH-PVN neurons which excite the OX-LH neurons stimulating wakefulness in mice [115] further highlights the diverse involvement of the CRH neurons and their CORT production in balancing homeostatic processes.

### 7.2. Melatonin

The hypothalamic-regulated hormone melatonin is synthesized in the pineal gland and is integral to sleep/wakefulness. Melatonin peaks during the dark phase in both diurnal and nocturnal organisms, whereas most hormones are expressed at opposite phases between nocturnal and diurnal organisms. This suggests that melatonin release is regulated by mechanisms upstream of the unknown biological diurnal/nocturnal switch. Control of melatonin release occurs via direct excitatory glutamatergic inputs from the SCN to the pineal gland. During the dark phase, direct GABAergic inhibitory signals from the SCN to the PVN inhibit the PVN to pineal gland projections [118,240]. Rhythmicity in melatonin provides essential regulatory control in metabolism; when melatonin synthesis is abolished by pinealectomy, glucose tolerance is impaired and blood glucose rhythmicity is completely lost under ad libitum feeding conditions [241,242]. Thus, the melatonin-mediated regulation of plasma glucose levels is controlled by a delicate balance of glutamatergic and GABAergic pre-autonomic hypothalamic inputs. The daily rise in plasma glucose is produced by inhibition of GABAergic activity at sympathetic pre-autonomic neurons of the PVN, which also increases hepatic glucose output [243]. Altogether, melatonin acts as a key integrator of circadian rhythms and energy metabolism through its effects on the hypothalamus and the periphery.

### 7.3. Gut-Derived Polypeptides

Gut-derived polypeptides have also been implicated in circadian rhythmicity. Apart from the previously discussed GLP-1 and FGF21 hormones, other peripherally-derived hormones also modulate metabolism in a hypothalamus-dependent manner. The gastrin-releasing peptide (GRP) is a mediator of food intake and locomotor activity, and can induce light-like resetting of the SCN [244]. Recently, microbes residing in the gut have been shown to be highly relevant circadian factors in metabolism. Studies have demonstrated that certain microbial taxa and their secretions exhibit diurnal oscillations [245,246,247,248]. Additionally, the timing of food intake and chronodisruptions such as jet lag and shift work, can alter the abundance and functions of gut microbes [248,249]. Most interestingly, germ free mice which lack gut microbiota, actually display altered SCN and hepatic transcriptional rhythms, particularly in core clock genes and metabolic pathways [248]. While the gut microbe interaction with the clock machinery is still under investigation, these studies suggest an important relationship that may influence overall energy metabolism. Collectively, rhythmic cross-talk between the extra-SCN hypothalamus and the periphery is critical for metabolic homeostasis.

## 8. The Role of Intrinsic Clocks of the Hypothalamus in Obesity

The intrinsic molecular clock of individual cells drives rhythmic tissue-specific functions, while being entrained by a variety of neuroendocrine, metabolite, hormonal, electrical and temperature signals [148]. A large body of evidence supports the strong role of the clock in energy homeostasis. One of the first demonstrations of the clock being involved in obesity came from a study in 2005, demonstrating that *Clock* mutant (*Clock*^Δ19^) mice express accelerated hyperphagia, obesity and glucose intolerance when challenged with a high-fat diet (HFD) [250]. A number of circadian mutant rodent models show similar metabolic defects [17,251] and human epidemiological studies also support this association [18,252,253]. Circadian disruption through chronic jet lag, shift work, or other means has serious ramifications on human health. Epidemiological studies on shift workers reveal a predisposition to acquiring Type II Diabetes, obesity, cancer, cardiovascular issues and even Alzheimer’s disease or other neurological diseases [18,254]. Acute effects of shift work include disrupted sleep–wake cycles and altered meal timing, which is considered to desynchronize the peripheral metabolic clocks and is exacerbated by hormonal imbalance of key satiety signals like leptin and ghrelin [255,256]. A short-term study conducted on humans in attempt to stimulate the deleterious effects of jet lag or shift work entailed exposure to a light–dark cycle lengthened to a 28 h period [257]. Notably, participants displayed impaired glucose tolerance and hypoleptinemia, underscoring the immediate effects of circadian disruption on energy metabolism [257]. In short, rotational shift and night shift workers are predisposed to deleterious long-term effects on their metabolic, cardiovascular, neurological and psychological health [257,258,259].

### 8.1. Time-Restricted Feeding

While the timing of light exposure has robust control over physiological functions and behavior, the timing of food intake also plays a crucial role as well. Time-restricted feeding (TRF) offers a novel non-pharmacological treatment that shortens the window of energy intake to 8–12 h without inadvertently restricting total caloric intake. Preclinical and animal studies clearly demonstrate that TRF can prevent metabolic disease even in mice fed a HFD [260]. Though some studies suggest that TRF activates OX-PVN neurons [261] at the molecular level, TRF strengthens the otherwise dampened rhythms of clock genes, improves mTOR, AMPK and CREB signaling, reduces adiposity and alters the liver metabolome [260,262]. When TRF is applied during the rest phase, the phase of clock gene expression in peripheral tissues reverses, as does the phase of satiety hormones such as leptin, ghrelin and insulin, leading to weight gain and hepatosteatosis [263]. Using a rotating light cycle to mimic shift work, chow-fed mice show altered phases of insulin and CORT secretion, while HFD mice under rotating light have complete loss of rhythmicity in hepatic lipogenic gene expression [264]. Simply delaying feeding time by 4 h elicits a shift in peripheral clocks and increases weight gain in mice fed HFD [265]. Thus, TRF can actually reverse the deleterious side effects of obesity [266,267]. Even when only adhering to TRF on the weekday and following ad libitum feeding on the weekends in HFD fed mice, the beneficial effects of TRF are still seen [266,268]. At the hypothalamic level, TRF has been shown to restore disrupted locomotor activity in mice with lesioned DMH or jointly lesioned DMH and SCN [269]. Interestingly, hypothalamic leptin sensitivity is improved when TRF is aligned to the daily rhythms of leptin [270]. MC4R neurons in the ARC are highly involved in the regulation of the melanocortin system, which has been demonstrated to mediate the benefits of TRF on energy balance [271]. When the timing of food intake is misaligned with the individuals’ intrinsic clock, as is seen in shift workers or chronically jet-lagged individuals, long-term health ramifications exist.

### 8.2. The Influences of Diet on the Hypothalamic Clocks

Diet is also capable of remodeling circadian rhythms behaviorally, transcriptionally, and metabolically [22,260,272,273]. For example, feeding mice a ketogenic diet or overexpressing the FGF21 (whose production is stimulated by a ketogenic diet) augments locomotion during the daytime and results in a phase advance in mice [183]. Mice placed on standard HFD have a profound reorganization of their circadian gene expression and metabolite production, in several metabolic tissues, including liver, adipose tissue, and the CNS [22,272,273]. However, hypothalamic PER2::LUC rhythms in the SCN, DMH or ARC appear unaltered under HFD [91]. Is it possible that other hypothalamic regions are affected, or that the *Per2* locus itself is protected from nutrient challenge in the hypothalamus? The so called “Western diet” feeding across 16 weeks in mice results in attenuated neuropeptide expression of Npy, Pomc and AgRP [274]. Interestingly, these ARC neuropeptides are also altered in hypothalamic cell lines derived from global *Bmal1*-KO mice [274]. The prevalent saturated fatty acid in Western diet known as palmitate is capable of stimulating *Npy* expression [274]. However, palmitate exposure disrupts *Bmal1* and *Nampt* expression, the latter an integral enzyme of the NAD^+^ salvage pathway, which interacts with the core circadian clock, to induce neuroinflammation in hypothalamic neurons [275]. These findings highlight a mechanistic link between *Bmal1* expression and the effects of Western diet on rhythmic expression of energy related hypothalamic neuropeptides. The sensitivity of circadian timekeeping to unhealthy diets or stress is further supported by studies showing that HFD feeding blunts CRH-PVN neuron responsiveness to nutritional challenges and stress [276]. Typically stimulation of these neurons elicits their rapid activation; however, clamping CRH-PVN neurons at either high or low levels of activation appears to mimic the same blunting of responsiveness induced by HFD [276]. In both these models, blunting of CRH-PVN neurons results in reduced diurnal rhythmicity in feeding and energy expenditure [276]. AgRP-ARC neurons also display blunted inhibitory activity while under HFD [277]. The PVN, DMH, VMH and ARC have direct innervations to the ANS, some of which terminate in adipose tissue. These extensive projections allow hypothalamic control of WAT and BAT activity [278]. An important leptin-mediated feedback loop between the brain and adipose tissue has been shown to be important for long-term energy balance in mice. Specifically, the disruption of circadian rhythms through chronic jet lag is sufficient to induce leptin resistance independent of obesity risk factors [229].

Interestingly, the PVN has also been uncovered as an influential mediator of food selection. An intriguing study by Okamoto et al. 2018 demonstrates that activation of AMPK-regulated CRH-PVN neurons drives the selection of carbohydrates over fat in mice under a restricted feeding paradigm [279]. Altogether, this discovery that fasting may simulate AMPK to activate a subset of CRH-PVN neurons to induce preference for carbohydrates lays the foundation for further elucidation of the relationship between obesity, diet and the circadian clock.

### 8.3. Sex-Specific Differences

Circadian differences exist between sexes, which may extend to circadian regulation within specific hypothalamic nuclei. For example, recent research revealed that men and women placed in either circadian-aligned or circadian-misaligned conditions across 3 days displayed different physiological changes [280]. Specifically, women expressed circadian misalignment by changes in their energy homeostasis through decreased leptin and increased ghrelin levels [280]. On the other hand, men show increases in hedonic appetite, specifically for carbohydrates [280]. Comparing adolescent girls and boys under similar social jet lag, researchers found that a late chronotype is associated with increased adiposity in girls, but not boys [281]. These studies are two of several that underscore sex-specific differences in the expression of circadian misalignment, suggesting that studies may need to take into account sex when attempting to understand the mechanisms by which chronodisruption alters energy balance and circadian alignment.

## 9. The Role of Intrinsic Clocks of the Hypothalamus in Neurological Diseases and Disorders

A growing body of literature is addressing the mechanisms by which chronodisruption might predispose one to, or exacerbate, existing Alzheimer’s disease (AD), Parkinson’s disease and other neurological diseases [282,283,284]. Table 1 presents a summary of neurological diseases that are associated with rhythmic hypothalamic nuclei.

### 9.1. Alzheimer’s Disease, Neuroinflammation and the Disruption of Sleep–Wake Cycles

One major contributor to the pathogenesis of AD is neuroinflammation although its role in AD is not entirely known. However, accumulating evidence points to microglial and astrocytes whose regulation of CNS response to injury through pro or anti-inflammatory signals have been strongly implicated [285,286,287]. It is postulated the Aβ plaques stimulate astrocytes and microglial to secrete pro-inflammatory cytokines, chemokines, and reactive oxygen species whose chronic presence dysregulate the immune response that furthers neurodegeneration. Both astrocytes and microglia contain intrinsic clocks that control their morphology, internal processes and contribute to regulation of the sleep–wake cycle [288,289,290]. Circadian impairment in microglia and astrocytes has been implicated in the neuroinflammatory responses that progress AD [291,292,293]. One such study found that by disrupting BMAL1 in microglial using a synthetic REV-ERB agonist, an increased inflammatory phenotype appeared in microglial of the amyloid precursor protein knock-in (*App*-KI) mouse model. Moreover, cognitive tests on these 2-month-old *App*-KI mice treated with the REV-ERB agonist showed microglial activation and cognitive decline [291]. Implicating circadian disruption in microglial induces chronic neuroinflammation, which exacerbates AD.

A widely acknowledged symptom in patients with AD-associated dementia or PD is the disruption of sleep–wake cycles. A possible disconnect may exist between the rhythmic oscillations in the SCN and the regions involved in sleep–wake cycle homeostasis such as the LH, VMH, and DMH. However, pathological changes in the SCN of individuals with AD further implicate the potential role of the circadian clock in disease progression [294,295,296]. Increasing evidence points to several extra-SCN hypothalamic regions for the early stages of AD development. Notably, plaques and neuron fibrillary tangles have been observed in the PVN and LH of AD patients [295,297]. Although cognitive decline and dementia are the characteristic hallmarks of AD pathology, they are now recognized as manifestations during late stages of AD [298]. Chronic stress can induce tau pathology, neurodegeneration, and learning impairments in a transgenic mouse model of AD [299]. Treatment with the CRH receptor 1 antagonist prevents these deleterious effects of chronic stress, indicating that CRH production in the PVN mediated by the HPA axis may contribute to AD pathology [299]. However, studies examining circulating cortisol levels in relation to AD show mixed findings. Thus, further investigations are needed to understand the interactions among stress, circadian rhythms and AD pathology.

A study in 2015 revealed that Aβ plaques induce post-translational degradation of the circadian clock interactor and co-regulator CREB, as well as BMAL1, resulting in disruption of *Per2* expression [300]. Interestingly, amyloid levels are shown to peak during the day and decrease during sleep; however, when sleep is disrupted the amyloid levels measured by either cerebral spinal fluid or positron emission tomography generally show an increase [301,302,303]. In addition, when circadian genes are deleted in a mouse AD model, the mice develop more Aβ plaques than mice without the deleted clock genes while also displaying disrupted amyloid rhythms [301]. At the clinical level, a 2017 study reported a significant dysregulation in *Bmal1* expression was associated with early AD [304]. These data suggest a role for circadian genes and circadian rhythmicity in AD pathogenesis.

### 9.2. Sundowning Syndrome

A very interesting behavioral pattern in AD patients is the manifestation of Sundown Syndrome or “sundowing.” AD patients with sundowning express temporally-dependent behaviors of agitation, aggression, and/or confusion in the afternoon and evening hours [305]. Interestingly, a higher rate of sundowning is reported during the winter months when sunlight exposure is often limited [306]. Melatonin treatment for individuals with sundowning appears to reduce agitated behaviors [307]. The most studied and clinically used therapy is light therapy for sundowning patients, although, in some cases, the SCN may be too degenerated for light therapy to be effective [308]. One study reported that a non-invasive 40 HZ light flicker was sufficient to ameliorate the AD-associated rhythmic disruption in mice [309]. Sundowning severity increases as AD progresses, and light is sometimes recommended during the evening hours to reduce associated agitation. Interestingly, insomnia and hypersomnia are also positively associated with the degree of sundowning symptoms, suggesting a potential corollary factor of sleep fragmentation [310]. The consistent expression of agitation, aggression and/or confusion at a particular time point in the day strongly indicates a disruption of circadian function in the CNS, most likely the hypothalamus. Todd et al. 2018 identified a neurocircuit where the SCN projects to the SPVZ, whose neurons are most active during the early rest phase and innervate the VMH region [311]. Disruption of GABAergic activity in the SPVZ results in a time-dependent increase in aggressiveness in the mice, offering a potential neural circuit involved in sundowning [311]. The VMH region is also associated with the regulation of fear and anxiety, making it a candidate for involvement in sundowning-associated behaviors [312,313]. Further exploration will likely revolve around the rhythmicity of the SCN and its hypothalamic counterparts to elucidate the underlying neurocircuit involved in sundowning.

### 9.3. Seasonal Affective Disorder

This link between our intrinsic clocks and emotional state is not unique to AD or sundowning symptoms; other psychological disorders also have circadian-dependent properties. For example, seasonal affective disorder (SAD) is a type of depression that occurs most commonly during the fall/winter seasons [338]. A reduction in light and changes in the photoperiod length during the fall/winter seasons is presumed to trigger the depressive behaviors characteristic of SAD [339]. Treatment for SAD relies predominantly on phototherapy, in which patients are exposed in the morning to either bright white light or infrared light [327,337]. The finding that SAD prevalence varies depending on the latitude, increasing at higher latitudes where the days are shorter during the fall/winter time, further corroborates the relation between light and emotion [340]. While the underlying mechanism behind SAD is unknown, many consider the clock-regulated hypothalamus to be a prime suspect.

Some mechanistic insights into circadian links to SADs have been revealed in preclinical and human studies both. *Bmal1*-specific knockdown in the SCN results in a depressive anxiety-like phenotype accompanied by disrupted CORT rhythmicity [341], highlighting a circadian hypothalamic mechanism contributing to emotion. Seasonal changes in hypothalamic gene expression and hormonal activity have been observed in Siberian hamsters [342]. fMRI imaging in SAD patients during exposure to blue light has revealed enhanced responsiveness in the posterior hypothalamus to auditory emotional stimuli, suggesting this region’s involvement in SAD [329]. In addition, the dysfunction of hypothalamus-related functions in SAD individuals is reflected by alterations in sleep, feeding and metabolism [338,343]. Appetite is markedly increased in SAD patients, particularly for carbohydrate-rich foods, and weight gain, lethargy, sleepiness and sleep issues such as insomnia also arise more frequently in SAD patients [343,344]. These issues implicate potential roles for the PVN, due to its involvement in carbohydrate preference, and the LH, for its regulation of sleep–wake cycles, in SAD pathology. Moreover, seasonal differences in diurnal cortisol release in SAD patients further implicates a role for the PVN and the HPA axis [336]. The changes in the sleep–wake cycle of SAD patients is also suggestive of hypothalamic OX signaling. Recently, a study highlighted a potential SAD pathway comprised of the SCN, OX-LH neurons and the downstream dorsal raphe nucleus [345]. The quantity of OX hypothalamic neurons was reduced in SAD animals kept in dim light to simulate winter light exposure [346]. Furthermore, exposure to normal light in combination with an OX antagonist led SAD mice to a depressive phenotype [346], suggesting that OX neuron signaling is involved with the depressive behaviors associated with SAD.

### 9.4. Mood Disorders

Thus, while SAD behavior appear to have a light-dependent origin mediated through a rhythmic hypothalamus, dissimilar neurological phenotypes, including mania, have also been correlated to light-dependent hypothalamic activity. Mania is associated with bipolar disorder (BD) in which individuals commonly vacillate between episodes of depression and mania. BD is often accompanied by disrupted rhythms in the sleep–wake cycle, activity, appetite and hormonal secretions. Moreover, bouts of mania often follow a cyclical, even seasonal pattern, suggesting an underlying circadian mechanism. *Clock*^Δ19^ mice actually express a similar behavioral profile to mania. Treatment with the mood stabilizer lithium, a therapeutic for BD patients, eliminates mania behavior in the *Clock*^Δ19^ mice, revealing a novel role for CLOCK in mood regulation [347]. Intriguingly, the *Clock*^Δ19^ mice have augmented dopaminergic activity in a region downstream of the hypothalamus, the ventral tegmental area (VTA) [347]. The expression of CLOCK in the VTA of *Clock*^Δ19^ mice often eliminates the manic phenotype [347]. Furthermore, the knockdown of Clock expression in the VTA results in greater depressive behaviors although less hyperactivity [348]. This is perhaps not surprising, as cocaine-induced dopaminergic surge results in a profound reprogramming of circadian gene expression in the striatum, a processes partially dependent on the dopamine 2 receptor and pro-inflammatory peroxisome protein activator receptor gamma (PPARγ) activity [349].

The VTA is not the only non-hypothalamic region recently uncovered for its rhythmic role in emotion. In schizophrenia, which is characterized by psychotic behaviors, transcript analysis in post-mortem brain tissues schizophrenia patients reveal altered rhythmic gene expression in the dorsolateral prefrontal cortex [330]. The hypothalamic region is still likely involved in schizophrenia as other studies have found increases in hypothalamic volume [350] and attenuated AVP mRNA expression in the hypothalamus [351]. As studies venture beyond the hypothalamus, it is evident the complexity of emotional regulation involves numerous regions. Changes in brainstem substructure volumes have recently been associated with seasonal changes in photoperiod length suggesting a novel role for the brainstem in SAD [352]. A fascinating neurocircuit study by An et al. 2020 uncovered a circadian gated subcortical pathway for the induction of depressive-like behaviors in response to light at night exposure in mice [353]. Electrophysiological studies have demonstrated that the photosensitive ipRGCs, which project to the dorsal perihabenular nucleus (dpHb), have greater excitability during the nighttime. Projections from the dpHb terminate on the nucleus accumbens to mediate depressive-like behaviors induced by light exposure during the nighttime [353]. This novel circuit potentially underlies the higher propensity for shift workers to develop MDD and anxiety disorders [354].

Mitochondrial metabolic dysfunction may also underlie the behavioral changes associated with mood disorders such as BD and schizophrenia. In mice with forebrain-specific mitochondrial mutations, the behavioral phenotype resembles a manic episode and is accompanied with disrupted circadian expression [355]. The interlocking of intracellular metabolism with circadian machinery via the NAD^+^ salvage pathway suggests a direct link whereby circadian disruption may mediate or worsen metabolic dysfunction, leading to poor cellular health or even cell death. Most likely, however, the metabolic disruptions result in altered neural plasticity and hormone production. For example, CLOCK and NAD^+^ regulation of SIRT1 diurnally antagonize CREB-mediated transcription of tyrosine hydroxylase [356]. In *Clock*^Δ19^ mice, this diurnal regulation is absent, resulting in elevated *TH* levels during the daytime [356]. *TH* is the rate-limiting enzyme for the synthesis of dopamine, a hormone very likely involved in emotion. Thus, the interactions between core clock mechanisms, metabolism and emotions are more widespread and intertwined than localized circuitry between the SCN and other hypothalamic nuclei.

## 10. Treatments and Therapeutics

Though the circadian mechanisms controlling hypothalamic-driven energy intake and metabolism are still emerging, new data suggest that circadian-enhancing small molecules might be a road to improved metabolism and rhythmic activity in the context of nutrient excess and metabolic disease. Examples include the BMAL1-modulating small molecule, Nobiletin (NOB), which acts as the ligand for the *Bmal1* locus-activating transcription factor ROR [357]. NOB can boost rhythmicity in energy intake and energy expenditure and inhibit many of the obesity-associated peripheral co-morbidities, such as fatty liver disease and WAT expansion [357,358,359]. Moreover, in Type II Diabetes, characterized by dysfunction of the pancreatic insulin-secreting β cells, treatment with NOB can enhance the circadian amplitudes of β cell-mediated insulin secretion in a *Bmal1*-dependent manner [360,361]. Though the application of such circadian modulators in the context of neurological diseases is in its genesis, it will be important to know to what extent fortification of the internal clock can reverse or prevent neurological diseases and their symptoms. Certainly, internal rhythms reprogram with age [314,362,363,364] and reinforcement of “youthful” rhythmicity through overexpression of SIRT1 in the SCN of the hypothalamus, or by augmenting NAD^+^-mediated activity can delay the aging phenotype [315,316]. On a practical level, adherence to a relatively rigid sleep–wake and feeding cycle that is in line with the active phase should at least be strong considerations for preventing metabolic and neurological diseases. Further studies will hopefully reveal the extent to which circadian modulators can be used for treating these diseases successfully. However, preclinical studies are beginning to give us hope that the circadian clock might be exploited to this end.

## Figures and Tables

**Table 1:** Hypothalamic nuclei rhythms and associated neurological diseases and disorders.

Neurological Disease/Disorder	Rhythmic Regions Involved	Evidence	References
Alzheimer’s Disease (AD)	SCN	AB plaques SCN neuron and glial degeneration Alterations in mitochondria and synaptic degeneration Decrease in VIP-SCN neurons in presenile female AD patients Increase in activated astrocytes and reduction in vasopressin and GABA production Abnormal rhythmicity in locomotion and body temperature. Bright light during LD cycle reduces AB accumulation in AD mouse model	[291,293] [292] [314] [315] [316]
	PVN	AB plaques and NFT present Increased cortisol levels Altered CRH levels Neuronal loss, alterations in mitochondria and loss of dendritic branching and spines	[317] [318] [319] [320]
	LH	High density of AB plaques and NFT Genetic variations in orexin signaling receptors are possible risk factors MCH levels are elevated and correspond to cognitive decline Increased orexin levels are correspond to increasing sleep fragmentation	[294] [321] [322] [323]
Sundowning Syndrome	SCN	Expression of symptoms is light dependent	[302]
	PVN	Simulation of this region leads to aggression	[324]
	VMH	Increased salivary cortisol levels	[325]
	LH	Stimulation of this region leads to aggression Agitation corresponds to higher levels of orexin correspond to increased fragmented sleep	[324] [326]
Seasonal Affective Disorder (SAD)	SCN	SCN-Bmal1-KD mice display depressive behaviors SCN→OX-LH neurons→dorsal raphe nucleus	[327] [328]
	PVN	CORT dysregulation Appetite increase and carbohydrate preference	[329] [311]
	LH	Disrupted sleep/wake cycle and insomnia SCN→OX-LH neurons→dorsal raphe nucleus	[311] [328]
	Brainstem	Seasonal and photoperiod alterations in volume	[330]
Bipolar Disorder (BD)	LH	Sleep disturbances are predictive of mania or depressive episodes Reduced plasma OX levels Kindling of the LH induces manic activity in mice	[312] [331] [332]
	PVN	HPA axis is implicated to be involved Increased cortisol appears to precede manic episode Neuronal loss	[27,333] [334] [335]
	VTA	Clock expression in VTA of Clock^Δ19^ mice eliminates mania-like behaviors	[336]
Schizophrenia	dlPFC	Altered gene rhythmicity	[337]

## Abbreviations

βOHB	β- hydroxybutyrate
AD	Alzheimer’s Disease
ADIPOQ	Adiponectin
AgRP	Agouti-Related Peptide
AMPK	5′Adenosine Monophosphate-Activated Protein Kinase
ANS	Autonomic Nervous System
App-KI	Amyloid precursor protein knock-in
ARC	Arcuate Nucleus of the Hypothalamus
AVP	Arginine Vasopressin
BAT	Brown Adipose Tissue
BBB	Blood–Brain Barrier
BD	Bipolar Disorder
BDNF	Brain-Derived Neurotrophic Factor
BMAL1	Brain Muscle Arnt-Like Protein 1
Camk2a	Calcium/Calmodulin-Dependent Protein Kinase II Alpha
CART	Cocaine And Amphetamine-Regulated Transcript
CB1R	Cannabinoid Receptor 1
CCG	Core Clock Genes
CCK	Cholecystokinin
CLOCK	Circadian Locomotor Output Cycles Protein Kaput
CK1δ	Casein Kinase 1 Delta
CK1ϵ	Casein Kinase 1 Epsilon
CNS	Central Nervous System
CORT	Corticotrophin
CREB	Camp Response Element-Binding Protein
CRH	Corticotrophin-Releasing Hormone
CRY	Cryptochrome
CRY1	Cryptochrome 1
CRY2	Cryptochrome 2
CTP1c	Carnitine Palmitoyltransferase 1c
dlPFC	Dorsal Lateral Prefrontal Cortex
DMH	Dorsal Medial Hypothalamus
dpHb	Dorsal Perihabenular Nucleus
eCB	Endocannabinoid
FAA	Food-Anticipatory Activity
FEO	Food-Entrainable Oscillator
FGF21	Fibroblast Growth Factor 21
GABA	Gamma-Aminobutyric Acid
GLP-1	Glucagon-Like Peptide 1
GRP	Gastrin-Releasing Peptide
HFD	High-Fat Diet
HPA	Hypothalamic–Pituitary Axis
ipRGCs	Induced Pluripotent Retinal Ganglion Cells
JNK	C-Jun N-Terminal Kinase
Kiss1	Kisspeptin 1
Lep	Leptin
LH	Lateral Hypothalamus
MCH	Melanin-Concentrating Hormone
MC4R	Melanocortin Receptor 4
MPOA	Medial Preoptic Area
mTOR	Mammalian Target of Rapamycin
mTORc1	Mammalian Target of Rapamycin Complex 1
NAD^+^	Nicotinamide Adenine Dinucleotide
NAM	Nicotinamide
NAMPT	Nicotinamide Phosphoribosyltransferase
NMN	Nicotinamide Mononucleotide
NOB	Nobiletin
NPAS2	Neuronal PAS Domain Protein 2
NPY	Neuropeptide Y
NR1D1	Nuclear Receptor Subfamily 1 D Member 1
NR1D2	Nuclear Receptor Subfamily 1 D Member 2
NREM	Non-Rapid Eye Movement
NTS	Nucleus Tractus Soilaris
OT	Oxytocin
OX	Orexin
PACAP	Pituitary Adenylate Cyclase-Activating Polypeptide
PB	Parabrachial Nucleus
PDYN	Prodynophin
PER	Period
PER1	Period 1
PER2	Period 2
PNS	Parasympathetic Nervous System
POMC	Proopiomelanocortin
PPARγ	Peroxisome Protein Activator Receptor Gamma
PVN	Paraventricular Nucleus
p75NTR	Neurotrophin Receptor P75
REV-ERBα	Nr1d1
REV-ERBβ	Nr1d2
RF	Restricted Feeding
RHT	Retinohypothalamic Tract
RORs	Retinoic Acid Receptor-Related Orphan Receptors
SAD	Seasonal Affective Disorder
SCN	Suprachiasmatic Nucleus
SF1	Steroidogenic Factor 1
SIM1	Single-Minded Homolog 1
SIRT1	Sirtuin 1
SPVZ	Subparaventricular Zone
TRF	Time-Restricted Feeding
TRH	Thyrotropin-Releasing Hormone
TrkB	Tropomyosin Receptor Kinase B
TRPM2	Transient Receptor Potential Cation Channel, Subfamily M, Member 2
TF	Transcription Factor
TTLF	Transcription–Translation Feedback Loop
VLPO	Ventral Lateral Preoptic Area
VTA	Ventral Tegmental Area
